# The Diversity of Trematodes in *Myotis* Bats (Chiroptera, Vespertilionidae) from the Samarskaya Luka (European Russia)

**DOI:** 10.3390/ani13233738

**Published:** 2023-12-03

**Authors:** Nadezhda Yu. Kirillova, Alexander A. Kirillov, Victoria A. Vekhnik

**Affiliations:** Laboratory for Biodiversity, Institute of Ecology of the Volga Basin of the Russian Academy of Sciences, Samara Federal Research Center of RAS, Togliatti 445003, Russia; nadinkirillova2011@yandex.ru (N.Y.K.); ivavika@rambler.ru (V.A.V.)

**Keywords:** bats, Digenea, *Myotis* spp., Middle Volga region, Samarskaya Luka National Park

## Abstract

**Simple Summary:**

Different species of bats often inhabit the same territories but have differences in diet. Bats become infected with parasitic worms such as trematodes when they feed on insects. Fluke diversity is determined by bats’ dietary breadth. The purpose of our research was to study trematode diversity in five *Myotis* species inhabiting Samarskaya Luka National Park. In the 2005–2007 period, we studied parasitic worms in bats and found 11 trematode species. One parasite species, *Prosthodendrium cryptolecithum*, was recorded for the first time in Russia. Only three fluke species were revealed in all studied bat species. Our study shows that trematode species diversity is higher in *Myotis daubentonii* and *M. dasycneme*. The trematode fauna of *M. brandtii*, *M. nattereri*, and *M. mystacinus* is less diverse. Our results confirm the similarity of habitats and diets among the five *Myotis* species and show weak competition among bats for food items.

**Abstract:**

Various bat species often occupy the same habitats. Cohabitation should induce different preferences in spatial or trophic components of the bat ecological niche to reduce their competition. This determines the differences in the trematode fauna of *Myotis* spp. The purpose of our research was to study the biodiversity of trematodes in syntopic populations of five *Myotis* species in the Samarskaya Luka National Park. In the 2005–2007 period, we studied 867 bat specimens via the methods of complete helminthological dissection. In total, 11 trematode species from the families Plagiorchiidae (*Plagiorchis koreanus, P. mordovii*, *P. muelleri*, and *P. vespertilionis*), Pleurogenidae (*Parabascus duboisi*), and Lecithodendriidae (*Prosthodendrium ascidia*, *Pr. chilostomum*, *Pr. cryptolecithum*, *Pr. hurkovaae*, *Pr. longiforme*, and *Lecithodendrium linstowi*) were found in five *Myotis* species. Only three trematode species, *P. koreanus*, *Pr. chilostomum*, and *P. duboisi*, are common to all studied *Myotis* spp. *Prosthodendrium cryptolecithum* was recorded for the first time in Russia. Trematode species diversity is higher in *Myotis daubentonii* and *M. dasycneme*. The trematode fauna of *M. brandtii*, *M. nattereri*, and *M. mystacinus* is less diverse. The determining factor in the infection of bats with trematodes is feeding on semi-aquatic insects, possible second intermediate hosts of the parasites. The infection of bats with flukes occurs at different levels of host (*Myotis* spp.) abundance as a result of the realization of the main bat trophic relationships. Our results confirm the data that the ecological niches of the five *Myotis* species partially overlap. Analysis of the trematode fauna in *Myotis* spp. showed that, in the Samarskaya Luka, there may be weak competition for food items among bats.

## 1. Introduction

Recently, more and more attention has been paid to the study of groups of co-inhabiting and even taxonomically close species of bats. In such communities, rather complex and contrasting relationships develop between their members [1,2,3,4,5]. Various bat species often occupy the same habitats. In this case, the principle of competitive exclusion can be applied, according to which species with the same ecology cannot live in the same space. Co-habiting species should exhibit different preferences for some components of the ecological niche, either spatial or trophic, in order to reduce their competition [6].

The territory of European Russia is inhabited by 27 species of bats. The bat fauna of the Middle Volga region includes 16 species [7,8,9,10]. Among them are five species of the genus *Myotis* (Chiroptera, Vespertilionidae): the whiskered bat *Myotis mystacinus* (Kuhl, 1819), Daubenton’s bat *Myotis daubentonii* (Kuhl, 1817), pond bat *Myotis dasycneme* (Boie, 1825), Brandt’s bat *Myotis brandtii* (Eversmann, 1845), and Natterer’s bat *Myotis nattereri* (Kuhl, 1817). All studied bats are sedentary species, wintering in underground shelters, caves, and abandoned adits [2,9]. Among the sedentary bat species inhabiting the Samarskaya Luka, the populations of *M. brandtii* and *M. daubentonii* have the highest abundance [2].

Despite relative similarities in habitat preferences, various *Myotis* spp. have significant trophic and chorological differences [7,11,12]. The lifestyle differences, particularly their feeding habits and spatial distribution, affect the trematode fauna of *Myotis* spp. In this regard, studies on the helminth fauna of syntopic *Myotis* species and, especially, their flukes, are of particular interest.

Studies of helminths in various bat species of the genus *Myotis* have been carried out in many European countries [13]. The most studied helminth fauna is of *M. mystacinus*; its diversity has been studied in Poland, Austria, Italy, France, Switzerland, the Czech Republic, Slovakia, Romania, Moldova, Ukraine, Belarus, Georgia, and Azerbaijan. In this bat species, 27 species of parasitic worms, including 16 trematode species, have been registered in Europe and the countries of the former USSR [13,14,15,16,17,18,19,20,21,22,23,24,25,26,27,28,29,30,31,32,33,34,35,36].

The helminth fauna of *M. dasycneme* has been studied in the Czech Republic, Poland, and Hungary [18,20,21,23,37]. Data on the helminths in *M. daubentonii* were obtained in France, Germany, Italy, Norway, the Czech Republic, Slovakia, Hungary, Poland, Belarus, Ukraine, and Turkey [16,18,20,21,23,37,38,39,40,41,42,43,44]. The parasitic worms in *M. nattereri* have been studied in Switzerland, Austria, Spain, the Czech Republic, Slovakia, Hungary, Poland, and Belarus [15,17,18,20,21,23,37,43,45,46,47].

Data on *M. brandtii* helminths in Western and Central Europe are known only from studies by Shimalov et al. [43,47]. The scarcity of data on the parasitic worms of *M. brandtii* in Europe is probably due to the fact that *M. mystacinus* and *M. brandtii* began to be distinguished as independent species only in the last 40 years [7,8]. Thus, data from helminthological studies of *M. mystacinus* apparently refer to both sympatric chiropteran species.

There are known data about trematodes in *Myotis myotis* Borkhausen, 1797, from Spain, Switzerland, Austria, Germany, the Czech Republic, Slovakia, Hungary, and Poland [14,18,20,21,23,38,45,46,48]. Trematodes in *Myotis emarginatus* (E. Geoffroy, 1806) have been studied in France, Austria, the Czech Republic, and Poland [18,20,21,23,40,45].

There is little information about trematodes, as well as about other helminths, of *Myotis blythii* Tomes, 1857, which have been studied in Slovakia, Hungary, and Serbia [18,48,49]; *Myotis capaccinii* Bonaparte, 1837, which have been studied in France, Italy, and Bulgaria [34,35,40,50]; *Myotis bechsteinii* Kuhl, 1817, which have been studied in Switzerland, the Czech Republic, and Ukraine [14,18,31,38]; and *Myotis aurascens* Kuzyakin, 1935, and *Myotis alcathoe* Helversen et Heller, 2001, which have been studied only in Turkey [44,51].

Recently, Frank and coauthors [13] presented a summary of parasites (including seven trematode species) in *Myotis* spp. inhabiting European countries. Unfortunately, this review did not include some data on parasites of bats from Western and Central Europe and the former USSR.

There are very few works containing data on helminths in *Myotis* spp. in Russia. The first report of parasitic worms in *M. mystacinus* was presented by Sten’ko et al. [52], where three species of trematodes were found in Crimea. Podvyaznaya [53,54] studied one species, *Allassogonoporus amphoraeformis* (Mödlinger, 1930), from *M. brandtii* in the Voronezh Nature Reserve. Demidova and Vekhnik [55] studied trematodes in *M. mystacinus* and *M. brandtii* from the Samarskaya Luka, in which two and nine trematode species were identified, respectively. Five species of trematodes were found in *M. brandtii* from Karelia [56]. Gulyaev and co-authors [57] provided data on one studied specimen of *M. brandtii* from the Magadan Oblast, in which only *Plagiorchis* sp. was found. Previously, we studied the helminths in *M. dasycneme* and *M. daubentonii* from Mordovia, where three and six trematode species were identified in bats, respectively [58,59]. Our previous works, devoted to parasites of vertebrates from the Middle Volga region, include information about flukes in all five chiropteran species [60,61,62,63,64].

Thus, the aim of our present research was to study the biodiversity of trematodes in syntopic populations of five *Myotis* species from the Samarskaya Luka (European Russia).

## 2. Materials and Methods

**Figure 1:**
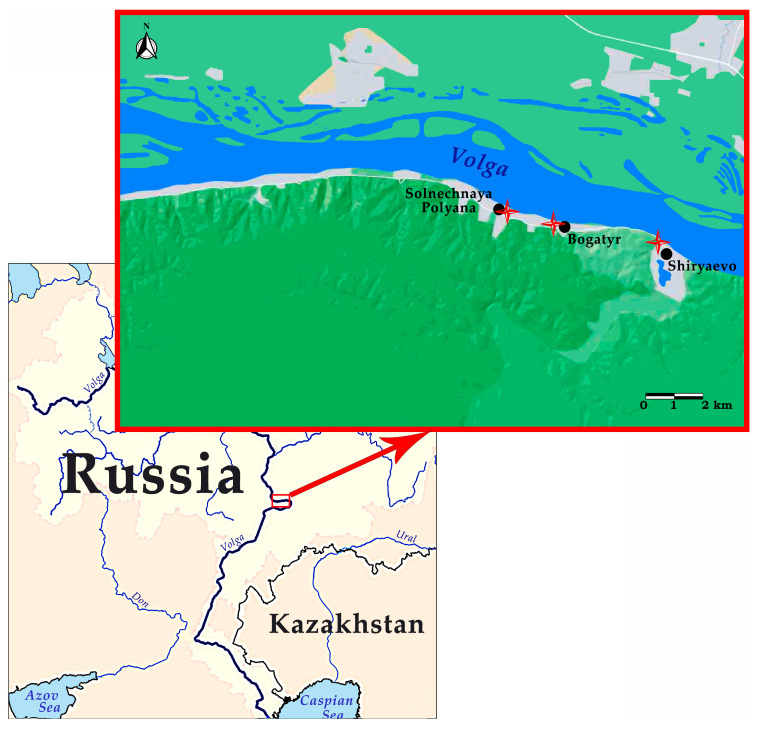
Map of bat trapping sites in the Samarskaya Luka National Park. Red crosses on the map indicate the trapping sites.

The material for this research was collected during our own field studies on bat ecology and helminths in the territory of the Samarskaya Luka National Park (Samara Oblast, European Russia), which were conducted in the 2005–2007 period. The study area was the coastal zone of the Volga River in the northern part of the Samarskaya Luka (Samara Oblast). Bats were studied at three trapping stations of the National Park near Solnechnaya Polyana, Bogatyr and Shiryaevo villages (Figure 1).

In total, we studied 247 *M. brandtii*, 262 *M. daubentonii*, 135 *M. dasycneme*, 125 *M. mystacinus*, and 98 *M. nattereri*. Bats were caught with mist nets at night. We used the common method of placing the net between two vertical sticks, which were 6 m long telescopic fishing rods [65]. We also studied bats that died of natural causes in their wintering sites.

Bats were examined using complete helminthological dissection [66,67]. Trematodes were collected from bats and fixed in 70% ethanol for further investigations. Trematodes were stained with aceto-carmine, dehydrated in a graded ethanol series (70–96%) and cleared in clove oil. The parasitic worms were then mounted in Canada balsam. Trematode species were identified according to Zdzietowiecki [20,23], Khotenovsky [24], Sharpilo and Iskova [31], Tkach et al. [41], and Skrjabin and Antipin [68].

To characterize the helminth infection of *Myotis* bats, the following indices, which are generally accepted in parasitology, were used: prevalence (*P*, %), mean abundance (*MA*), intensity range (*I*, specimens), and mean intensity (*MI*) [69,70]. To determine the species diversity of trematodes, Shannon (*H*’) and Shannon evenness (*E*) indices were calculated. The significance of differences between Shannon index values was measured using Student’s *t*-test. The degree of similarity between the trematode faunas of *Myotis* spp. was revealed using the Jaccard (*C_J_*) (qualitative data) and Sørensen (*C_N_*) (quantitative data) similarity indices [71]. The degree of similarity was assessed as low (0–0.33), medium (0.34–0.66), and high (0.67–1).

The dominance of species in the parasite community was determined using the Palia-Kovnatsky dominance index (*D*) [72]. Trematode dominance groups were as follows: 10–100, dominants; 1–10, subdominants; 1–0.001, adominants. The Mann–Whitney (*U*) test was used to compare the total infections of *Myotis* spp. and assess the significance of differences in infection with individual trematode species. A dendrogram of similarity of helminth faunas of *Myotis* spp. was obtained using the unweighted pair group method with arithmetic average (UPGMA) and the Morisita index as a distance measure in PAST 2.17 [73]. To standardize bat sample size, we used the species richness index (bootstrap estimator), which predicts the number of helminth species not included in collections [74,75]. The taxonomy of flukes was provided according to Fauna Europaea (http://www.fauna-eu.org/, accessed on 3 August 2023) [76]. Trematode voucher specimens qwee stored in the parasitological collection of the Institute of Ecology of the Volga River Basin of the Russian Academy of Sciences (Togliatti, Russia). Accession numbers are No. 332–338 (*P. vespertilionis*), 373–379 (*P. koreanus*), 322 (*P. muelleri*), 392–396 (*P. mordovii*), 231, 232, 238 (*Pr. ascidia*), 167, 260–262 (*Pr. chilostomum*), 179, 180, 256 (*Pr. cryptolecithum*), 289, 290 (*Pr. hurkovaae*), 205, 310 (*Pr. longiforme*), 202, 203, 217 (*P. duboisi*), 370 (*L. linstowi*).

## 3. Results

A total of 11 species of trematodes were revealed in five bat species from the Samarskaya Luka National Park (Table 1).

**Figure 2:**
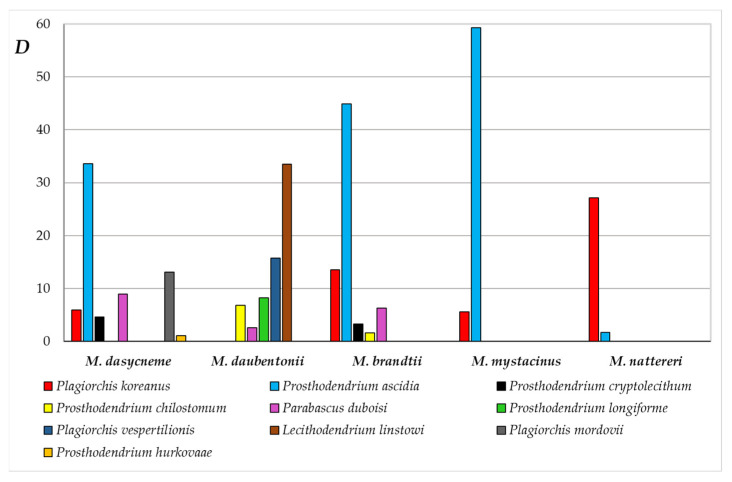
Dominant and subdominant species of trematodes in *Myotis* bats from the Samarskaya Luka (*D* – the Palia–Kovnatsky dominance index).

The greatest richness of the trematode fauna was found in *M. brandtii*, including 10 trematode species (Table 1). The total infection of *M. brandtii* with flukes reached 100%, *MA* = 50.4. According to the Palia–Kovnatsky dominance index, two species dominated in the trematode fauna of *M. brandtii*: *Pr. ascidia* (*D* = 44.9) and *P. koreanus* (13.5). The group of subdominants included *P. duboisi* (6.3), *Pr. cryptolecithum* (3.3), and *Pr. chilostomum* (1.6) (Figure 2).

Eight species of trematodes were revealed in *M. daubentonii* and *M. dasycneme*. The total infection in both species was 100%; the abundance index was 64.2 and 67.6, respectively.

The trematode fauna of *M. daubentonii* are dominated by *L. linstowi* (33.5) and *P. vespertilionis* (15.7), while *Pr. longiforme* (8.2), *Pr. chilostomum* (6.8), and *P. duboisi* (2.6) are subdominants. The trematode fauna of *M. dasycneme* are dominanted by *Pr. ascidia* (33.6) and *P. mordovii* (13.1); subdominants are *P. duboisi* (8.9), *P. koreanus* (5.9), *Pr. cryptolecithum* (4.6), and *Pr. hurkovaae* (1.1).

In *Myotis mystacinus*, as well as in *M. nattereri*, six species of trematodes were found. The total infection of *M. mystacinus* with flukes was 60%, *MA* = 12.8. Among trematodes identified in *M. mystacinus*, *Pr. ascidia* is dominant (59.3), while *P. koreanus* (5.6) is subdominant. The total infection of *M. nattereri* compared to other *Myotis* bats is low and amounted to only 32%, and the *MA* was 2.6. In the trematode fauna of *M. nattereri*, the dominant is *P. koreanus* (27.1), the subdominant is *Pr. ascidia* (1.7). The remaining four species in the trematode fauna of *M. nattereri* are adominants.

In the trematode fauna of *Myotis* bats, the structure and number of dominant and subdominant species are different (Figure 2). For all five *Myotis* species, no common dominant or subdominant trematode species was identified. Only two species of trematodes, *P. koreanus* and *Pr. ascidia*, are the dominant or subdominant for four bat species, and *P. duboisi* for three *Myotis* species (Figure 2).

Only three of the eleven trematode species were found together in all five bat species: *P. koreanus*, *Pr. chilostomum*, and *P. duboisi* (Table 1). *Prosthodendrium ascidia* was found in four species of bats. *Plagiorchis vespertilionis*, *P. mordovii*, *P. muelleri*, *Pr. hurkovaae*, and *L. linstowi* were each recorded in three host species. Two host species were noted for *Pr. cryptolecithum* and *Pr. longiforme* (Table 1).

Values of trematode species diversity in *Myotis* spp. from the Samarskaya Luka National Park are shown in Table 2.

The diversity indices of the trematode fauna are significantly higher for *M. daubentonii* and *M. dasycneme*, with the exception of the Margalef index values. The values of the Margalef index for these bat species are slightly lower than for *M. brandtii* and *M. nattereri* (Table 2). The trematode fauna of *M. brandtii* and *M. nattereri* are less diverse. The least diversity in the trematode fauna was observed in *M. mystacinus* (Table 2). This bat species has a significantly lower Shannon diversity, Shannon evenness, Margalef and Simpson evenness indices than other species (Table 2). The differences in the Shannon species diversity index among the trematode fauna of five chiropteran species are significant (*p* < 0.001), with the exception of the pair *M. daubentonii*—*M. dasycneme* (*p* > 0.05).

A comparison of trematode infection of five *Myotis* species using the Kruskal–Wallis test revealed significant differences (H = 425.6, *p* < 0.0001). A pairwise comparison of infection of different *Myotis* spp. using the Mann–Whitney test revealed significant differences in most cases (*p* < 0.0001), with the exception of the pair *M. daubentonii*—*M. dasycneme* (Table 3).

An analysis of the infections of bats with common trematode species using the Mann–Whitney test showed significant differences in infection with *Pr. chilostomum* (in all cases), *P. koreanus*, and *P. duboisi* (in most cases) (Table 3). Differences in the infection with *P. koreanus* in pairs *M. brandtii*—*M. dasycneme*, *M. mystacinus*—*M. nattereri*, *M. nattereri*—*M. daunentonii*, as well as in the infection of *M. mystacinus* and *M. nattereri* with *P. duboisi*, were not significant. There were no significant differences in the infection with *P. koreanus* in pairs *M. brandtii*—*M. dasycneme*, *M. mystacinus*—*M. nattereri*, *M. nattereri*—*M. daunentonii*, as well as in the infection of *M. mystacinus* and *M. nattereri* with *P. duboisi* (Table 3).

**Figure 3:**
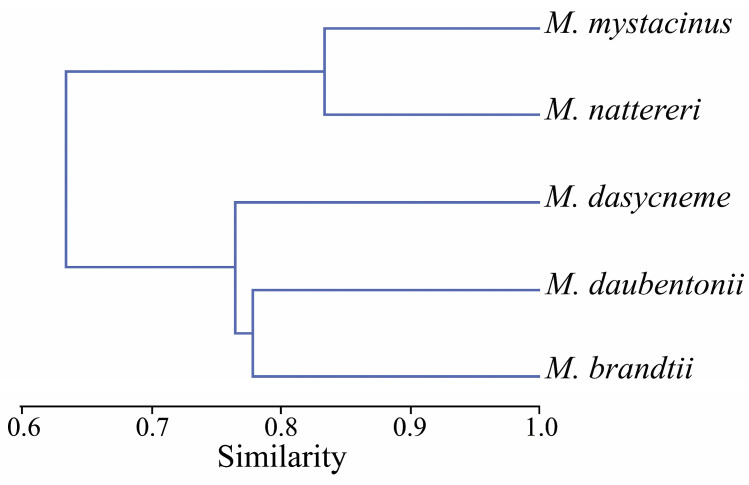
Similarity dendrogram obtained using Morisita index (UPGMA) for the trematode fauna of *Myotis* spp. from the Samarskaya Luka National Park. Cophen. corr.: r = 0.802.

We conducted a comparative analysis of the helminth faunas of *Myotis* species inhabiting the Samarskaya Luka. A similarity dendrogram of the parasite fauna of five bat species is presented in Figure 3.

A cophenetic correlation coefficient of 0.802 confirms the validity of the cluster. As a result of the cluster analysis, the considered *Myotis* species were divided into two clusters with the most similar helminth fauna (Figure 3).

Flukes parasitizing *M. mystacinus* and *M. nattereri* form the first cluster, in which the greatest similarity of trematode communities is observed (0.83). These species of bats differ maximally in trematode composition from the three other *Myotis* spp. thatare included in the second cluster (Figure 3).

The second cluster is formed by a clade of trematode faunas of *M. brandtii* and *M. daubentonii* (0.78) that is close to the trematode fauna of *M. dasycneme*, more similar to that of *M. brandtii* (0.78), and less similar to that of *M. daubentonii* (0.75) (Figure 3). The remaining pairs showed an average degree of similarity ranging from 0.57 to 0.63.

## 4. Discussion

Analysis of the trematode fauna of *Myotis* spp. from the Samarskaya Luka National Park showed that the species composition was the richest in *M. brandtii* (10 species). Slightly fewer trematode species were found in *M. daubentonii* and *M. dasycneme* (eight species each), and the smallest number of trematode species was found in *M. mystacinus* and *M. nattereri*—six species each (Table 1).

Analysis of the species diversity of trematodes in *Myotis* spp. showed that the trematode fauna are more diverse for *M. daubentonii* and *M. dasycneme*, despite the fact that a larger number of trematode species were found in *M. brandtii* (Table 1 and Table 2). The value of the Shannon diversity index for *M. brandtii* is lower, which is associated with the high abundance and dominance of one species in its trematode fauna, *Pr. ascidia* (Figure 2). At the same time, the Margalef index, which takes into account species richness and the total number of parasite specimens, is significantly higher in *M. brandtii*, from which the largest number of trematodes was collected (Table 2). The abundance and dominance of *Pr. ascidia* in *M. mystacinus* also leads to a significant decrease in the Shannon index. As a result, this species of bats has the lowest species diversity of the studied flukes (Table 2, Figure 2).

All trematode species were found in *Myotis* spp. at the mature stage and are host-specific parasites of bats. Three of the eleven recorded trematode species (*Pr. ascidia*, *Pr. cryptholecithum*, and *P. duboisi*) in bats in the study area were found only in *Myotis* spp. In addition to *Myotis* spp., other species of bats from the Samarskaya Luka also participate in the life cycles of eight more species of trematodes [60,61,63].

Infection of bats with trematodes occurs entirely when feeding on semi-aquatic insects, which serve as second intermediate hosts of these parasites. Thus, findings of *P. koreanus* and *P. vespertilionis* indicate the consumption of adult forms of Diptera, Ephemeroptera, Megaloptera, Trichoptera, and Odonata by bats [31]. The presence of *Pr. chilostomum* in the helminth fauna of *Myotis* spp. indicates that bats consume adults of Trichoptera and, possibly, Odonata [31]. The second intermediate hosts of *Pr. ascidia* are the chironomid larvae, such as *Chironomis plumosus* (Linnaeus, 1758) [31,77].

The life cycles of *P. mordovii*, *P. muelleri*, *L. linstowi*, *Pr. longiforme*, *Pr. cryptolecithum*, *Pr. hurkovaae*, *P. duboisi*, and *P. lepidotus* are currently not studied. Probably, the second intermediate hosts of these trematodes, like other bat trematodes, are insects that develop in the aquatic environment [31].

Trematodes are parasites with a complex life cycle, including a change of hosts. Accordingly, the trematode fauna of *Myotis* spp. depends on the presence/absence of various first (freshwater gastropods) and second (semi-aquatic insects) intermediate hosts at each site. In turn, the species composition of trematodes and their infection of intermediate hosts are determined by the diversity and abundance of final hosts (bats), which ensure the release of trematode eggs into the external environment.

According to Smirnov and Vekhnik [12], among *Myotis* spp., in terms of relative abundance index, *M. daubentonii* (10.3%) is the most numerous in the Samarskaya Luka; *M. dasycneme* (7.5%) and *M. brandtii* (6.5%) are slightly less common. *M. mystacinus* (2.3%) and *M. nattereri* (0.2%) are rare in the study area. Analysis of the trematode fauna of bats showed that the most abundant species, *M. daubentonii*, does not have the largest number of trematode species (eight) but the same number as in *M. dasycneme* (Table 1). *Myotis brandtii*, inferior in relative abundance to *M. daubentonii* and *M. dasycneme*, nevertheless has the largest number of trematode species (10). The least abundant species, *M. nattereri*, has the same number of trematodes as *M. mystacinus* (six species each) (Table 1). When analyzing the infection of bats with trematodes, significant differences were established, which confirm that the highest parasitic load of trematodes is carried by *M. daubentonii* and *M. dasycneme*, to a lesser extent than *M. brandtii*, and the lowest parasitic load on *Myotis* spp. is revealed in *M. mystacinus* and *M. nattereri* (Table 1 and Table 3).

It should be noted that the infection of bats with flukes occurs at different levels of host abundance (*Myotis* spp.) as a result of the realization of the main trophic relationships between chiropterans. The presented differences in the trematode fauna of various *Myotis* species are mainly associated with preferences for certain food items. The widest feeding range was found in *M. daubentonii*, whose diet in the Samarskaya Luka includes 10 food items from nine orders of insects. The diet of *M. dasycneme*, *M. brandtii*, and *M. mystacinus* is slightly smaller and contains nine food objects from eight orders of insects [12]. *Myotis nattereri* also has nine food items, but from seven orders of insects [12].

Despite the similar composition of the diet, the proportion of various food items of the five bat species is not the same [12]. Thus, the main diet of *M. daubentonii* and *M. dasycneme* consists of Trichoptera, Lepidoptera, and Diptera. Coleoptera are somewhat less represented in the diet of these two species, and among the food items for *M. daubentonii* there are also representatives of the orders Hemiptera and Homoptera. Insects of other orders are rarely found in the diet of *M. daubentonii* and *M. dasycneme* [12].

The diet of *M. brandtii* is dominated by Lepidoptera and Diptera; Coleoptera are rather less represented; insects of the orders Trichoptera, Neuroptera, Homoptera, and Hymenoptera are rarely found [12]. In *M. mystacinus*, the main diet components are Lepidoptera; Trichoptera are less represented, and Coleoptera, Homoptera, and Diptera are rare. The diet of *M. nattereri* is dominated by Lepidoptera. Members of Trichoptera, Coleoptera, Diptera, Hymenoptera, and Homoptera are present in the diet in equal proportions [12].

The identified differences in the infection of bats with common trematode species can be explained by the different proportions of food items (second intermediate hosts of trematodes) in the diet of *Myotis* spp.

The diversity of the trematode fauna of bats also depends on the number of hunting grounds that bats visit. Visiting more hunting sites expands the bats’ diet, which ultimately increases the likelihood of their infection with various species of trematodes.

*Myotis* spp. show unequal preferences for different types of hunting grounds. Among bats, the largest number of hunting sites was found for *M. brandtii*, *M. daubentonii*, and *M. dasycneme* [78]. *Myotis nattereri* prefers to hunt in only one type of hunting sites, whereas *M. mystacinus* has two types of hunting grounds [78]. Our identification of a greater number of trematode species in *M. brandtii*, *M. daubentonii*, and *M. dasycneme*, compared to that in *M. nattereri* and *M. mystacinus*, confirms these data on hunting preferences (Table 1).

In addition, *Myotis* spp. occupy different spatial niches when hunting sites overlap [78]. Thus, *M. daubentonii* and *M. dasycneme* hunt above the water surface, *M. brandtii* feeds high in the tree crowns, and *M. mystacinus* hunts immediately below (the middle and lower parts of the tree crowns). *Myotis nattereri* prefers to hunt in open confined spaces above the ground, such as forest edges, wide forest glades, and paths [78]. The association of different *Myotis* species with certain hunting grounds also affects the diversity and abundance of insects in the diet of bats. Ultimately, this determines the species composition and abundance of trematodes in *Myotis* spp.

A comparative analysis of the trematode fauna of five *Myotis* species inhabiting the Samarskaya Luka National Park showed a significant similarity in the composition of trematodes in different bat species (Figure 3). This is due to their similar lifestyle and feeding on semi-aquatic insects, which serve as second intermediate hosts of flukes. Thus, the finding of three common trematode species in bats (*P. koreanus*, *Pr. chilostomum*, and *P. duboisi*) indicates that they feed on the same food items (insects)—the second intermediate hosts of these trematode species. Our data confirm the partial overlap of spatial and trophic niches of various *Myotis* spp. and their division according to food preferences and hunting grounds.

## 5. Conclusions

Eleven trematode species were found in five species of *Myotis* spp. from the Samarskaya Luka National Park. The trematode *Pr. cryptolecithum* was recorded in bats of Russian fauna for the first time.

Analysis of the trematode species diversity in *Myotis* spp. showed that the trematode fauna are more diverse in *M. daubentonii* and *M. dasycneme* (8 species each), despite the fact that a larger number of trematode species were found in *M. brandtii* (10). The lowest species diversity of trematodes was observed in *M. mystacinus*, while the number of trematode species noted in *M. mystacinus* and *M. nattereri* is the same (six species each). All *Myotis* species participate to varying degrees in the realization of the life cycles of trematodes on the territory of the Samarskaya Luka. However, abundant bat species (*M. daubentonii*, *M. dasycneme*, and *M. brandtii*) carry the main parasitic load and have both the highest trematode species diversity and high infection rates. Despite the relatively low abundance of *M. mystacinus* and *M. nattereri* and their low infection with trematodes, these species are also involved in the distribution and preservation of the abundance of the parasites.

The determining factor in the infection of bats with trematodes is feeding on semi-aquatic insects (second intermediate hosts). Therefore, the infection of bats with trematodes occurs at different levels of host abundance (*Myotis* spp.) as a result of the realization of the main trophic relationships.

The diversity of the trematode fauna of *Myotis* spp. is associated with the number of hunting sites that bats visit. Various *Myotis* species visiting the same hunting grounds and consuming the same food items (but in different proportions) have an average degree of similarity in the trematode fauna. The revealed differences in the trematode fauna of *Myotis* spp. are associated both with the food preferences of various species and with the variety of hunting grounds they use.

Our parasitological results confirm the data on the partial overlap of the ecological niches of the five *Myotis* species in spatial and trophic components. An analysis of the trematode fauna of *Myotis* spp. showed that, in the territory of the Samarskaya Luka, there may be weak competition for food items among bats due to their specialization and divergent habitats.

## Figures and Tables

**Table 1:** Trematodes in *Myotis* spp. from the Samarskaya Luka National Park.

Trematode Species	*M. brandtii*	*M. daubentonii*	*M. dasycneme*	*M. mystacinus*	*M. nattereri*
Family Plagiorchiidae					
*Plagiorchis koreanus* (Ogata, 1938)	81.4(1–111)10.3/8.4 ^1^	28.2(1–26)5.2/1.5	63.7(1–52)9.8/6.2	43.2(1–10)3.9/1.7	40.8(1–13)4.2/1.7
*Plagiorchis mordovii* (Schaldybin, 1958)	25.1(1–15)3.9/1.0	3.8(1–5)2.0/0.1	76.3(1–50)15.2/11.6	–	–
*Plagiorchis muelleri* (Tkach et Sharpilo, 1990)	24.4(1–9)2.8/0.7	–	–	13.6(1–6)2.3/0.3	10.2(1–5)2.0/0.2
*Plagiorchis vespertilionis* (Müller, 1780)	18.2(1–8)2.5/0.5	79.4(1–99)16.0/12.7	11.1(1–21)7.1/0.8	–	–
*Prosthodendrium ascidia* (Beneden, 1873)	87.5(1–178)29.6/25.9	–	74.8(3–225)40.6/30.4	72.0(1–51)14.6/10.5	12.2(1–5)2.9/0.4
*Prosthodendrium chilostomum* (Mehlis, 1831)	32.8(1–62)7.3/2.4	56.1(1–73)13.8/7.7	16.3(1–13)5.1/0.8	0.8(27)27.0/0.2	6.1(1–3)1.7/0.1
*Prosthodendrium cryptolecithum* (Zdzitowiecki, 1969)	39.7(1–70)10.6/4.2	–	53.3(1–94)10.9/5.8	–	–
*Prosthodendrium hurkovaae* (Dubois, 1960)	–	22.9(1–60)8.7/2.0	29.6(1–21)8.1/2.4	–	5.1(1–3)2.0/0.1
*Prosthodendrium longiforme* (Bhalerao, 1926)	27.5(1–9)2.9/0.8	66.8(1–110)11.6/7.9	–	–	–
Family Pleurogenidae					
*Parabascus duboisi* (Hurkova, 1961)	54.7(1–59)10.6/5.8	36.6(1–61)12.6/4.6	63.0(1–58)15.2/9.6	2.4(2–3)2.3/0.1	3.1(1–5)3.0/0.1
Family Lecithodendriidae					
*Lecithodendrium linstowi* (Dollfus, 1931)	9.7(1–31)8.8/0.9	76.7(1–125)36.1/27.7	–	1.6(1)1.0/0.02	–
Total	10	8	8(8.0) ^2^	6(6.6)	6(6.1)

Note: ^1^—prevalence of infection (*P*), in brackets—intensity range (*I*), after brackets—mean intensity (*MI*), beyond the line—mean abundance (*MA*); ^2^—in brackets—predicted number of species not included in collections due to insufficient host sampling.

**Table 2:** Values of trematode biodiversity indices in *Myotis* spp.

Index	*M. brandtii*	*M. daubentonii*	*M. dasycneme*	*M. mystacinus*	*M. nattereri*
Margalef index, *D_Mg_*	0.955	0.719	0.768	0.678	0.906
Shannon index, *H’*	1.555	1.584	1.592	0.616	1.127
Shannon evenness index, *E*	0.675	0.762	0.766	0.344	0.629
Simpson index, *d*	3.175	3.817	3.718	1.439	2.110

**Table 3:** Validity of differences in the infection of *Myotis* spp. with common trematode species.

Bat Species	*Plagiorchis koreanus*		*Parabascus duboisi*		*Prosthodendrium chilostomum*		Total Infection	
	*U*	*p*	*U*	*p*	*U*	*p*	*U*	*p*
*M. mystacinus*/ *M. brandtii*	7549.0	0.0001	7222.0	0.0001	10,540.0	0.0001	3895.0	0.0001
*M. mystacinus*/*M. daubentonii*	14,310.0	0.016	10,650.0	0.0001	7375.0	0.0001	2328.0	0.0001
*M. mystacinus*/*M. dasycneme*	5496.0	0.0001	3214.0	0.0001	7141.0	0.0001	1156.0	0.0001
*M. mystacinus*/*M. nattereri*	**6048.0**	**0.858**	**6084.0**	**0.760**	5740.0	0.013	2433.0	0.0001
*M. dasycneme*/*M. brandtii*	**14,840.0**	**0.073**	13,760.0	0.01	13,860.0	0.001	1234.0	0.0001
*M. dasycneme*/*M. daubentonii*	10,210.0	0.0001	12,500.0	0.0001	9814.0	0.0001	**17,330.0**	**0.7455**
*M. dasycneme*/*M. nattereri*	4296.0	0.0001	2551.0	0.0001	5970.0	0.027	69.5	0.0001
*M. brandtii*/*M. daubentonii*	13,430.0	0.0001	27,540.0	0.002	22,380.0	0.0001	24,650.0	0.0001
*M. brandtii*/*M. nattereri*	5873.0	0.0001	5738.0	0.0001	8847.0	0.0001	292.0	0.0001
*M. daubentonii*/*M. nattereri*	**11,390.0**	**0.057**	8419.0	0.0001	6124.0	0.0001	166.5	0.0001

Note: unsignificant differences are shown in bold (*p* ˃ 0.05).

## Data Availability

The data presented in this study are available on request from the corresponding author.
